# The Legal Regulation of Cybercrimes related to Character Assassination under the Jordanian and French Legislation

**DOI:** 10.12688/f1000research.177079.1

**Published:** 2026-03-05

**Authors:** Ashraf Al-Rai, Dina Imad, Maya Khater

**Affiliations:** 1 Collage of Law, Abu Dhabi University, Abu Dhabi, Abu Dhabi, United Arab Emirates; 2 Collage of Law, Abu Dhabi University, Abu Dhabi, Abu Dhabi, United Arab Emirates; 3 Collage of Law, United Arab Emirates University, Al Ain, Abu Dhabi, United Arab Emirates

**Keywords:** Digital Character Assassination; Criminal Jordanian law; French Legislation; Cybercrimes.

## Abstract

**Background:**

This study seeks to recommend amendments to the Jordanian Cybercrime Law No. 17 of 2023, especially regarding the character assassination crime. The research mainly deals with the current legal framework's ambiguities by clarifying the legal terms and conceptual boundaries associated with this type of crime. The researchers point out that the law needs to be revised in order to include the current digital communicational channels which sometimes become the places of defamation that cause the loss of moral and reputation, thereby lighting the present ways of moral and reputational harm in the digital world.

**Methods:**

The study adopts a desciptive approach to explore the concept of character assassination from the perspectives of law and theory. Furthermore, a legal analytical approach is employed in order to evaluate the applicable provisions of Jordanian law, especially Article 16 of the Cybercrime Law, through the interpretation of statutory language, the identification of gaps, and the assessment of its concordance with general principles of criminal law and international legal systems.

**Results:**

The research findings indicate that Jordanian statutes have clearly acknowledged the concept of moral character assassination which is quite the opposite of the approaches taken in some other legal systems. Yet, the prevailing law still does not give a clear picture on the issue of criminal liability. Who can be considered a perpetrator of such crimes is one of the unclear areas in particular. In addition, the law's relevance to digital and internet-based interactions is still not crystal clear, thus leading to uncertainty as regards both enforcement and legal interpretation.

**Recommendations:**

The research suggests amending the Jordanian Cybercrime Law, specifically Article 16, to make the law more certain and effective. It also recommends to add the word "moral" explicitly and to make the online and digital domains unambiguously referenced so that the law will be able to comprehend present-day reputational harm and adapt to current technological conditions.

## Introduction

The Jordanian legislator has introduced a new crime under the Article 16 of the Cybercrime Law No. 17 of 2023, describing it as “character assassination,” which is in fact the “moral assassination of character”. However, there is still no precise definition given in the law. After a thorough examinations of legal texts in this regard, no clear and explicit definition of the term character assassination could be identified, except the mention of its being close to other types of offenses already existent in Jordanian legislation and in some other countries' laws including France. In this context, comparative laws often categorize character assassination as a part of defamation, libel, spreading false information and rumor collection.

As a result, “character assassination” is viewed as a legally ambiguous offense par excellence, which lacks a precise meaning and definition. The term has a wide-ranging connotation, i.e., it refers to a person's moral level where reputations are injured. Such vagueness calls for a thorough study of the offense, its constituent parts, and its distinction from other crimes like defamation, libel, and the spreading of falsehoods that are under Article 15 of Cybercrime Law No. 17 of 2023. In addition to this, “character assassination” is an offense that is different in nature from the rest of the offenses in the Jordanian legal system, thus making it difficult to establish its material elements.

In Article 16, the aforementioned statute gives the following definition: “The dissemination of information, the attributing, or the assigning of acts to a person unjustly that could be classed as character assassination through the use of information networks, information technology, information systems, websites, or social media platforms shall result in imprisonment of not less than three months or a fine of not less than 5,000 Jordanian dinars and not more than 20,000 Jordanian dinars, or both penalties”.

On the other hand, the legal system in France regards moral character assassination as “moral defamation” while it still goes under the term “defamation” as per the penal code. The French Press Law of 1881 in its Article 29 defines defamation and libel as “every allegation or accusation that harms the honor or reputation of a person”. Public defamation has been subjected to punishment under Article 32 with fines and compensations for the party wronged among the penalties.

The advent of the internet has brought about the challenge of digital defamation. France enacted the Online Hate Law of 2020 with the purpose of controlling the online abuses while the United Kingdom set the Defamation Act 2012 which became the main citation for defamation cases in England and Wales. Yet, defamation laws in Scotland and Northern Ireland are different but still in agreement on the essential principles.

One of the main points against the Jordanian Cybercrime Law No. 17 of 2023 is that it does not properly separate moral character assassination from offenses like defamation, libel, and slander; this can be seen in the definitions provided in Article 2. The matter is serious enough to require a detailed analysis, and the research advises the Jordanian legislator to amend the law by clearly distinguishing between the relevant offenses and eliminating any ambiguities with regard to the terms mentioned. Moreover, there is a need for a proper definition of the two related terms “rumor dissemination” and “spreading false or fake news”.

The idea of “moral character assassination” was not recognized by early legal scholars, as it was more or less based on the exaggerated or partially true reports that were meant to portray a distorted picture of the target, which could be a person, a public figure, or an institution. Some define it as “death of a person's morality with the physical body still alive”, whereby a mental picture completely opposite to the person's reputation is created. For example, a person of integrity could be the victim of a financial scandal rumor, thus creating the impression of corruption and, consequently, the crime's impact would be greater.

Defining the term “character assassination” is not only a theoretical problem but also a practical problem to solve in the legal field, especially in the digital era when information spreads very quickly. In this context, the Jordanian Cybercrime Law No. 17 of 2023 is a landmark measure in overcoming contemporary challenges. Nevertheless, as already pointed out, the employment of ambiguous expressions such as “character assassination” might result in misunderstandings about the nature of offenses and create hurdles in the application of law.

In legal systems, the use of accurate terms is of utmost importance since the lack of definitions can lead to different interpretations. Therefore, it is very necessary for the Jordanian legislator to improve the intelligibility of legal definitions and to, in particular, indicate the differences between different acts that damage reputation. The act of providing unambiguous definitions will not only ease the process of legal classification but also protect individuals and society, and mark out clear limits within the legal text.

The investigation into character assassination has been done in terms of its legal definition and regulatory measures, which are discussed in two main parts, and finally, the conclusion is drawn along with suggestions based on the analysis of the legal provisions and their effectiveness in dealing with this offense.

The issue to be investigated in this research is to assess the Jordanian Cybercrime Law No. 17 of 2023 in terms of its capability to regulate the offense of character assassination, to provide a precise definition of the crime, and to make a clear the difference between character assassination and other crimes like defamation, libel, and slander, and the spreading of false information. Accordingly, the research raises the following fundamental questions:

• What is the crime of character assassination?
• What are the elements of the crime of character assassination?
• What are the shortcomings of the Jordanian Cybercrime Law regarding the regulation of character assassination?
• How does character assassination differ from defamation, libel, and the dissemination of false information?

## Methodology

This study adopts a descriptive approach for the exploration of character assassination, its legal and theoretical basis, manifestations, and implications. The case of character assassination is approached through the descriptive method to understand its conceptual aspects and to distinguish it from the adjacent concepts of defamation and libel. Besides, the research takes a legal analytical pathway to assess the current provisions in Jordanian law regarding this type of crime and particularly Article 16 of the Cybercrime Law. The legal analytical method involves a meticulous interpretation of the statutory text, identifying the critical components of the offense, and considering the law's purpose that underlies the provision. It also investigates possible legislative gaps or ambiguities, evaluating the extent to which the provision conforms with the fundamental principles of criminal law, such as legality, proportionality, and legal certainty, and its alignment with comparative and international legal standards.

## Results

### 1. The crime of character assassination

The Jordanian Penal Code does not offer a definition for the term “character assassination”, just like the French penal legislation, as pointed out in the study's introduction. This term became part of the Jordanian Cybercrime Law No. 17 of 2023 and is viewed as an “undefined” or ambiguous concept without any specific definition. Its use is generally for the purpose of representing the damage done to a person's ethical reputation.

The concept of moral character assassination was not known to early jurists, as its roots lie in the dissemination of exaggerated or partially truthful information aimed at presenting a distorted and false image of the intended target, whether a natural or legal person ( Icks & Shiraev, 2014).

Some define it as “killing a person morally while their body remains alive. This is achieved by creating a mental image that completely contradicts what the person is known for. For example, if a person is reputed for integrity, but rumors are spread accusing them of involvement in financial scandals, they become a victim of moral assassination, causing the public to view them as corrupt or immoral” ( Al-Amawi & Balas, 2024).

A ruling by the Jordanian Court of Cassation stated:

“In the context of character assassination, defamation, and distortion of the plaintiffs’ reputation after violating their dignity and personal and public freedoms, the defendant habitually spread false and fabricated rumors against the plaintiffs. They wrote, edited, and published materials in clear Arabic, which were false in substance, fabricated in form, and based on lies” ( AlOmran, Al-Rai, & Alhendi, 2024). “The defendant used written language as a tool for character assassination. The published materials and their style of presentation, including permitting their dissemination and using provocative language to attract attention, damaged the plaintiffs’ reputation, especially since internet readers tend to skim content. The allegations and claims made in these published materials were false and deliberately fabricated” ( Cassation Court, 2022).

A thorough examination of a number of judicial decisions in Jordan reveals that the majority of such cases are related to the crimes of defamation of public authorities. In the latest ruling issued by the Ma'an Magistrates Court in the southern part of Jordan, it is pointed out that: “The act of falsely attributing information to a person, which is considered to be character assassination in violation of Article 16 of the amended Cybercrime Law, the court holds that the writings that the defendant is said to have done consist of all the elements of defamation, slander, and insult but at the same time do not fall under the newly delineated scope of character assassination in the amended Cybercrime Law. As a result, the court proclaims the defendant innocent of this crime, as one of the elements of defamation, slander, and insult is already involved” ( *Magistrates Court*, 2024). The distinction between defamation and character assassination is underscored by this ruling, which however does not provide a clear definition of the latter. The phrase “character assassination” is included in Article 4 of the Jordanian Integrity and Anti-Corruption Law No. 13 of 2016, which declares:

The Commission aims to ensure adherence to the principles of national integrity and combat corruption through:

− Strengthening values and behavioural codes in public administration.
− Ensuring high-quality, transparent, and fair service delivery.
− Ensuring public administration compliance with governance principles, equality, and merit-based opportunities.
− Guaranteeing transparency in executive authority decisions and public access to information.
− Enforcing laws with transparency, achieving justice and equality.
− Providing a legal framework for holding decision-makers accountable.
− Receiving and addressing complaints via the law.
− Investigating financial and administrative corruption and taking legal action.
− Prosecuting corruption and preventing offenders from travel or accessing funds.
− Combating character assassination.

The term is also found in Article 68 of the repealed Civil Service Regulation, where the ethical duties of public service staff were illuminated. Furthermore, it is amply cited in Article 13 concerning the Economic and Social Corporation for Military Retirees and Veterans Regulation No. 13 of 2016 and in the Human Resources Management in the Public Sector Regulation No. 33 of 2023.

This indicates that character assassination, as intended by the legislator, is linked to corruption and public administration. We recommend revising Article 16 of the Cybercrime Law No. 17 of 2023 to define the legal meaning of this crime clearly and to remove ambiguity. We also agree with proposals to eliminate the inclusion of joint criminal participation in the previous provision, as it is already addressed in Article 27 of this law. Moreover, narrowing the gap between the minimum and maximum fines for character assassination penalties is advised ( Al-Shawabkeh, 2024).

### 2. The material element of the crime of character assassination

The material element of this crime lies in deliberately, and without justification, spreading, attributing, or ascribing actions to the victim that could lead to character assassination. Linguistically, the term “spread” is defined as “to mention or propagate something,” such as the phrase: “This is widespread news” meaning it has reached everyone and become common knowledge. The term “attribute” means to assign or relate something to someone, as in saying, “Person A attributed the statement to Person B” meaning they related it to them.

In the same respect, Article 16 of the Jordanian Cybercrime Law explicitly states:

"Anyone who deliberately spreads, attributes, or ascribes false actions to another person, or contributes to such behaviour through information networks, technology, information systems, websites, or social media platforms, intending to assassinate their character, shall be punished with imprisonment for no less than three months or a fine of no less than 5,000 dinars and no more than 20,000 dinars, or both penalties.”

The repetition of terms such as “spread” and “attribute” in the legal text is superfluous and unnecessary. The legislator could unify the terminology to improve the legal drafting, using terms like “spread” to describe disseminating information contributing to character assassination.

The statute that mostly governs defamation in France is the one dated July 29, 1881, which is related to press freedom. To constitute defamation, the act of harming or damaging someone's honor or reputation through publicizing or making statements or writings has to be proved as a material element of the crime. Such expressions can be written, oral, or be made known through the media or the internet. The public and the private defamation are the two categories that the French courts set apart, with the latter being restricted to a limited group of people, such as family, friends, and colleagues ( de Lamy, 2010).

Internet use in Jordan has become widespread and hence cyber crimes have increased tremendously. The statistics provided by the Cybercrime Unit of the Public Security Directorate indicate that the number of cyber crimes recorded in Jordan increased from 1,320 in 2013 to 16,027 in 2022, over a twelve-fold increase ( Maghaireh, 2024). Trends of this nature have been seen globally which have led to the conclusion of a cyber world that is under various threats, including the possibility of terrorist activities ( M. H. Khater, 2023).

Some legal scholars are of the opinion that only when the acts constituting the material element of the crime of character assassination will be committed repetitively can the crime be said to exist ( Capra et al., 2018). This interpretation originates from the fact that the legislator used the term “acts” in the plural rather than "an act" in the singular. We reject this interpretation, however. By saying “acts” the legislator is referring to the whole collection of prior factors ( Al-Rai, 2025).

Consequently, the material element of the crime is established whether the offense is committed through a single act, such as attribution or accusation against the victim, or through multiple acts.

### 3. The mental element of the crime of character assassination

The psychological aspect of the offense consists of criminal intent, which is a necessary requirement for the actor to purposefully perform an act that is targeted at exterminating the victim's character in a morally wrong way. The intent has to be present even if no harm is eventually done to the victim. The emphasis is placed on the action itself and not the damage caused, if the offender was by all means aware of the wrongful act, intended it, and knew it was illegal ( Awan, 2025). This is to say that the criminals are using the internet and other similar platforms unrightfully with complete understanding of the negative effects of their actions on people and society in general ( Khater, 2024a). The act must stem from the perpetrator's free and deliberate will. If the perpetrator refrains from completing the act or makes an unintended mistake, the crime is not established. However, the burden of proving such intent falls on the defendant, which can be challenging.

Accordingly, the mental element requires two main aspects:

− Awareness: Understanding the defamatory nature of the actions and the terms used.
− Will: The deliberate intent to utter or publish these statements, knowing they will harm the victim's reputation.

The French law considers the mental element to consist of both the intent to harm the reputation or dignity of the person affected and the awareness of the defendant that their actions are defamatory. The defendant must also intend to communicate them to others. Public defamation is subjected to more severe penalties than private defamation, with fines up to €45,000 and possible imprisonment in case of racially or religiously biased statements ( de Lamy, 2010). The material and mental elements of character assassination have been examined, and legal regulation of this crime requires a more in-depth consideration, which will be subject to research in the second section of this study ( Al-Rai, 2025).

### 4. The legal regulation of character assassination crime

The crime of character assassination in the Jordanian law has given rise to a number of problems, especially because of its likeness to other offenses in the Jordanian law, particularly the recent Cybercrime Law which has addressed libel, slander, and defamation expressly. Some laws in regard to electronic libel and slander use the word “defamation” which is derived from the Arabic word “ *shahara*” meaning to publicize or expose, which in turn implies becoming famous or widely known ( Ma’louf, 2005). In terms of definition, defamation is described as “the act of attributing certain material to the victim that causes hatred and contempt from others” ( Al-Zoubi, 2023).

The Jordanian Cybercrime Law explicitly addresses defamation in Article 20 Para A:

“Based on a complaint, anyone who uses an information network, information technology, information system, website, or social media platform to publish a recording, image, or video of something a person is keen to keep private, intending to defame, harm, or gain any benefit from doing so, even if obtained lawfully, shall be punished by imprisonment for no less than three months and a fine ranging between twenty thousand (20,000) dinars and forty thousand (40,000) dinars.”

According to Paragraph (B) of the same Article:

“Anyone who uses an information network, information technology, information system, website, or social media platform to alter, modify, or edit a recording, image, scene, or video of something a person is keen to keep private, intending to defame, harm, or gain any benefit from doing so, shall be punished by imprisonment for no less than two years and a fine ranging between twenty-five thousand (25,000) dinars and fifty thousand (50,000) dinars.”

In order to delve deep into the complexities of character assassination as a crime, we first have to look at the differences between this crime and other offenses specified in the Cybercrime Law in the first subsection. The second subsection will make suggestions for improving the legal regulation of character assassination crimes, and then we will wrap up with a collection of findings and recommendations.

### 5. Conceptual issues and legal distinctions between character assassination, libel and slander crimes

The distinction between moral character assassination, libel and slander crimes exists within the framework of Jordanian criminal legislation, where the Jordanian Cybercrime Law distinguishes between these concepts despite the fact that it does not provide a definition for moral character assassination. This is in stark contrast to other jurisdictions where such distinctions are not made, as moral character assassination, libel and slander are often considered more or less the same under a number of criminal legislations. However, the Jordanian legislator has defined libel and slander crimes explicitly in the Jordanian Penal Code No. 16 of 1960 and its amendments, as it will be detailed later.

Among the most common offenses related to the internet are crimes of libel and slander, and their misuse of technology is the main reason for this. The most important among them is the fact that the internet has countless places like social media, blogs, and websites where people can easily post harmful or offensive information. Consequently, the very nature of crime allows such information to be very rapidly and cheaply distributed to a large audience. Furthermore, it is sometimes the case that offenders are allowed to stay completely anonymous on the internet and this surely gives them the extra freedom of committing such crimes. The situation becomes more complicated by the unceasing monitoring and tracing activities of online content and the huge volume of information and data created every day. Thus, it becomes imperative to resort to the provisions provided in the Cybercrime Law in order to control these crimes.

It is noteworthy that even in States ranked as the top in the freedom of speech and expression category—like the US, Europe, and Australia—are applying very strict laws against defamation (which in many jurisdictions integrates libel and slander). Nevertheless, it should be noted that there is a wide difference between expressing an opinion that is in the public interest and the committing of a defamation crime ( Budiartha et al., 2018).

Both electronic libel and slander crimes involve material and moral elements, which will be discussed in two branches before delving into the exceptions to the use of libelous and slanderous expressions under the provisions of the Jordanian Penal Code in a third branch.

### 6. The material element of electronic defamation and insult crimes

Some scholas define defamation as attributing a specific matter to a person— even in the context of doubt or inquiry — in a way that would harm their honor and dignity or expose them to public hatred and contempt, whether or not the matter constitutes a punishable crime ( Heriyanto et al., 2024). This indicates that the material element of the crime of defamation is fulfilled when the perpetrator attributes a specific or defined matter to the victim, causing them to be subjected to public hatred, contempt, and harm to their dignity, honor, and reputation. Thus, electronic defamation is established through the act of attribution to the victim via electronic means, whether the perpetrator fabricated the claims themselves or merely repeated claims they had heard from others ( Al-Mashaqbeh, 2025). This is evident in paragraph (a) of Article 15 of the Jordanian Cybercrime Law No. 17 of 2023, which states: “Anyone who intentionally sends, re-sends, or publishes data or information via the internet, information technology, information systems, websites, or social media platforms that contain false news targeting national security, social peace, defamation, insult, or slander shall be penalized”.

Here, it is not required to explicitly mention the victim’s name; it suffices to provide indications pointing to the victim, as explicitly stated in the Jordanian Penal Code ( Al-Rai, AlOmran, & Al Ansari, 2025), which serves as the general legal framework. For example, the *Zarqa* First Instance Court, in its appellate capacity, ruled:

"Upon reviewing the case documents and evidence presented by the prosecution to prove the defendant's alleged crime, our court finds, based on the complainant's testimony (…), that they stated: He conducted a live broadcast on Facebook, cursing and insulting us, and posted on his Facebook page.’ However, upon reviewing the expert report which transcribed the recordings, there is no indication that the complainant was the target of the broadcast, as her name or description was not explicitly mentioned, nor were there any clues or signs suggesting the complainant was the subject of the post. Therefore, the defendant must be declared not responsible for the allegations" ( *Decision No. 802 of 2024*, 2024).

The material element is thus fulfilled through the acts of sending, re-sending, or publishing any kind of content, whether data—which refers to raw facts and figures that lack independent meaning without context, such as numbers, letters, symbols, or qualitative values ( Laudon and Laudon, 2019)—or information, which refers to processed or interpreted data that becomes meaningful and actionable, revealing relationships and insights when organized properly ( Stair, R., & Reynolds, 2020).

Defamation may also target a legal entity. Paragraph (b) of Article 15 of the law states: “Crimes mentioned in paragraph (a) of this article shall be prosecuted by the Public Prosecution without the need for a complaint or personal claim if directed against any state authority, official body, or public administration”.

Therefore, three conditions must be met to establish the material element of the crime of electronic defamation:

− Attribution of a matter: Assigning blame or insult to the victim.
− Specificity of the attributed matter: The content must consist of precise and defined expressions.
− Use of an electronic medium: The act of attribution must be carried out via electronic means, such as a computer, website, information system, or artificial intelligence platform.

The swift progression of technology and the digital world are behind the fact that these instances are not the only ones among many. If a defamation action is aimed at a legal entity that stands for a state power, a government body, or a public administrative unit, then the offender might be taken to court without the necessity of a formal complaint or personal claim. This shows the Jordanian legislator's rigorous method of preventing and punishing defamatory statements and ludicrous accusations against public institutions. Meanwhile, an insult does imply the same as the defamation but in a wider and less specific way. The same principles that govern the material element in defamation are also applicable to insult, thus, avoiding repetition.

### 7. The mental element in the electronic defamation and insult crimes

In order to catch the offender of electronic defamation or insult, it is necessary to the perpetrator's intention that the act would be computed as a crime, and along with that, their knowledge of the action and the possibility of its consequences to the victim's reputation, distress, and even the turning of the public opinion against him/her and subjecting him/her to hatred and contempt. This is clearly illustrated in paragraph (a) of Article 15 of the Jordanian Cybercrime Law No. 17 of 2023 which says: “Anyone who intentionally sends, re-sends or publishes data or information through the internet, information technology, information systems, websites or social media platforms that contain false news affecting national security, social peace, defamation, insult or contempt shall be punished”.

### 8. Components of criminal intent

− **Awareness:** The offender has to know the meaning and implications of the words or expressions they use and that these may hurt the victim's honor, dignity, or reputation or expose the victim to public contempt and hatred. This knowledge holds true in the same manner whether the victim is a natural person or a corporation. The knowledge in question must be real, not assumed. The offender has to be fully aware of the character of their acts, among which is that the statements imputed to the victim are disparaging, injurious to the victim's dignity, or likely to make the victim subject to hatred or contempt ( Child & Hunt, 2022).
− **Intent:** The intention of the offender must be to express or spread such statements, which would then lead to the desired result of making the victim publically hated or scorned. This is true whether the victim suffers any harm or not. It is the offence itself that is the basis of the crime’s establishment, and not the consequent damage. One scenario is that the perpetrator might claim that the victim made certain defamatory or insulting statements and would thus make the victim the target of disdain or contempt of a particular group of people—this could be a small circle or a larger audience. However, the victim's case could be such that the perpetrator's statements bear no truth, the victim is well-known in the city he belongs to, is part of the family or tribe, and as such, their audience recognizes the perpetrator's statements as false. In such a case, the victim might not suffer any actual harm. Nonetheless, the perpetrator is still liable for the act of defamation or insult, as the law considers the act itself and not its consequences ( Child & Hunt, 2022).

In our opinion, there is a mistake in the drafting of this article. The Penal Code, which is the general legal framework, treats defamation, insult, and contempt as separate offenses and provides for different penalties according to the crimes, whereas the article in question regards them as one single offense and applies the same punishment for all three. When applied to insult, the principles regarding the mental element in the crime of defamation equally apply to the crime of insult. To avoid repetition, we will not elaborate further.

### 9. Exceptions to the use of defamatory and insulting statements

While the Jordanian Cybercrime Law No. 17 of 2023, under Article 15, prohibits the use, transmission, retransmission, or publication of defamatory, insulting, or contemptuous statements through information networks, technology, systems, websites, or social media platforms. Such prohibitions ultimately aim to protect freedom of expression, preserving human dignity and maintaining social harmony ( Khater, 2024b). However, there are exceptions outlined in the Jordanian Penal Code, which serves as the general legislative framework. These exceptions, provided for specific cases, include:

− **Public Interest Criticism:** Defamatory or insulting statements may be permissible if they are true and serve the public interest. For such criticism to be lawful, certain conditions must be met:
  • The facts must be accurate.
  • The language used must be appropriate and proportionate.
  • The subject must provide societal value or benefit.
− **Official Publications:** Defamatory or insulting statements are exempt from criminal liability if they are included in:
  • Government or parliamentary publications.
  • Official documents or records, in accordance with Article 198(2) of the Penal Code.
− **Disciplinary Contexts:** Statements directed at individuals subject to military or police discipline are permissible if:
  • They pertain to the individual's conduct as part of such discipline.
  • They are made by a superior officer with authority to comment on such matters.
− **Judicial Proceedings:** Statements made during judicial proceedings, whether by the judiciary, lawyers, or witnesses, are exempt provided:
  • The publication is not prohibited by the court.
  • The trial is not confidential to ensure it does not interfere with the investigation process.

The Amman Court of First Instance, in a case bearing the number 2243 of the year 2002, had the example of a case where a journalist was not found liable of defamation. The court's justification was that the content that was published touched upon issues of public concern related to government financial and administrative irregularities that had already been questioned in the parliament and reported by the media. The complainant was not the article's target but was mentioned in their official capacity, thus the act was not a criminal one.

− **Parliamentary Announcements:** Defamatory or insulting statements made as part of accurate information disclosed in parliamentary sessions are exempt.
− **Reproduction of Published Material:** If a defamatory or insulting content represents an accurate reproduction, summary, or excerpt of material previously published and exempt from liability, its publication is also exempt.

### 10. Constitutional protection and legal boundaries

The Jordanian Constitution guarantees every citizen the right to express their opinion through speech, writing, images, or other means. The state is obligated to protect this right, provided the expression adheres to legal provisions. This underscores the balance between protecting individual rights and maintaining lawful boundaries for freedom of expression. In cases where defamatory or insulting language falls within these exceptions, such publications are not deemed criminal under the Penal Code. However, any use of such expressions must strictly comply with the conditions outlined in law to avoid abuse and ensure alignment with constitutional guarantees.

### 11. Proposed solutions for regulating the legal framework of moral character assassination crimes

The Jordanian Cybercrime Law has a lot of shortcomings in regulating crimes like digital character assassination through various platforms, social media, and modern media. It is clear that this crime is still so to say “unclear” to the Jordanian legal system. It overlaps with other crimes that are defined in laws such as the Penal Code, the Press and Publications Law, the Jordanian Journalists' Syndicate Law, and other acts related to the dissemination of information through digital channels ( Al-Zoubi, 2023).

To address these shortcomings, especially those related to digital platforms and modern media, we propose several measures to regulate moral character assassination crimes in Jordanian legislation, as follows:

− **Clarifying the Term “Moral Character Assassination” in Cybercrime Law**

This can be done through amending the provisions of the Cybercrime Law to explicitly define and delineate the legal concept of moral character assassination. It is also recommended that the legislator provides a comprehensive and precise legal definition to ensure clarity and consistent application.

− **Establishing Specific Penal Provisions for Media Platforms**

A special penal framework tailored for media platforms shall be introduced to achieve two primary goals:

• Promote freedom of opinion and expression by prohibiting the imprisonment of journalists and reducing financial penalties.
• Raise awareness among media professionals about the repercussions of moral character assassination crimes and the importance of refraining from actions that harm reputations.

This measure is especially significant given that many journalists and media professionals lack adequate legal knowledge in the field of journalism and media.

− **Updating and Revising Legal Provisions**

The current laws are to be evaluated in order to provide strong protection for the people from harm to their reputation. Also, a precise separation between the crimes of moral character assassination and the similar acts like defamation, slander, and contempt will be drawn.

− **Public Awareness Campaigns**

The use of public awareness campaigns is really important in making the public understand the distinctions between moral character assassination and some related offenses, but at the same time they are setting the limits that distinguish this crime from legitimate opinion, criticism, and freedom of expression.

− **Training Programs for Journalists and Media Professionals**

Defamation and moral character assassination impact the media and the public, so legal and ethical issues should be clarified in training programs for journalists and media professionals. These programs would not only support the implementation of the Cybercrime Law but also protect the right to free opinion and expression as stated in Article 15 of the Jordanian Constitution. This article guarantees that every Jordanian can express his/her views without fear, provided the expression is within the law's bounds.

The Jordanian law can more effectively regulate moral character assassination offenses through introducing these measures, thus also making the protection of personal reputations as an aspect of the freedom of expression right. The focus of these crimes is usually the media and press. One has to consider that the issue does not stem from the media institution's structure; rather it is the question of who is the publisher that poses the problem, a journalist or an activist on digital platforms. If the person is a journalist and commits the offense using their digital platform, such as Facebook, Twitter, or any other platform, then he or she will face the penalties based on the “Press and Publications Law” instead of the general legal rules.

However, some criminal law scholars argue that there is no need for a specific legal provision criminalizing these actions, as they are already covered by general laws, such as the “Criminal Code” and the “Jordanian Cybercrime Law”. On the other hand, some believe that only the provisions of the “Press and Publications Law” should apply to journalists and media professionals, regardless of the media institution they work for, with emphasis on financial penalties and the exclusion of custodial sentences. Here, a journalist is defined as a “member of the Jordanian Press Syndicate” as outlined in the definition of a journalist in the Jordanian Press Syndicate Law and the “Press and Publications Law”.

In cases where the perpetrator is not registered with the Jordanian Press Syndicate, which serves as the regulatory body for the profession, the provisions of Article 18 of the Syndicate Law may apply. This article stipulates that:

"A. Non-journalists, or journalists not specified in Article 9 of this law, are prohibited from sending correspondence to foreign newspapers or advertising themselves as journalists or using any phrase that implies such a role. Advertising, publishing, and distribution offices are also prohibited from adding any words or phrases to their titles, brochures, or advertisements that imply this role, unless they are licensed to publish press publications. B. The Syndicate shall issue press cards in accordance with its records. C. Any person who violates the provisions of paragraph (A) above shall be punished by a fine not less than two hundred dinars and not more than five hundred dinars, or imprisonment for a period not less than one month and not more than three months, or both penalties, along with an order to remove the violation. The penalty shall be doubled in case of repeat offenses."

The researchers believe that amending the “Press and Publications Law” to include all actions related to the journalist’s duties and imposing financial penalties for violations strengthens press freedom on one hand, and ensures journalists are subject to the “Press and Publications Law”, which is a special law for the journalistic and media profession, on the other hand. This should apply to journalists in the legal sense.

In the case of non-membership in the Syndicate, general laws should be applied, such as the “Criminal Code” the “Cybercrime Law” the Telecommunications Law, and any law that criminalizes such actions, as they would then fall outside the scope of crimes related to media and press, regardless of the form, whether traditional or non-traditional media.

## Conclusion

This study has addressed the legal regulation of the crime of defamation of personality through modern media and digital platforms in Jordanian criminal legislation, compared to French legislation. It has analyzed both the Penal Code and the Cybercrime Law and has reached several conclusions and recommendations in this field. Jordanian law has the uniqueness of dealing with the crime of defamation of personality in the Cybercrime Law of Jordan. Nevertheless, the lawmaker has not at all pointed out who the doer is and has not given an outright mention of whether the crime is restricted to digital platforms, social media, or modern media in general. On the other hand, the Jordanian Legislature, as usual, has not given a clear-cut definition of the crime. However, the French legislation has the opposite view: it is more elaborate and structured offering unambiguous definitions of defamation, slander, insults, and emotional distress, thus underlining the relative obscurity and the absence of conceptual clarity in the Jordanian legal system.

The paper recommends changing Article 16 of the Jordanian Cybercrime Law to indicate without any doubt the party who is responsible for the crime of defamation of personality. Besides that, the study urges the Jordanian legislator to make an open statutory definition of this crime in the Cybercrime Law and to use the word “moral” in order to make it clear that the intention behind the crime is moral defamation and not physical harm thus preventing any confusion especially in translation. The study lastly advocates for borrowing the French legislative style while making amendments to the relevant sections of the Jordanian Cybercrime Law or the Jordanian Penal Code.

## Data Availability

No datasets were generated or analysed during the current study, as the research is based on doctrinal legal analysis of legislation, case law, and secondary legal sources.
