# Supplementary material for: The Legal Regulation of Cybercrimes related to Character Assassination under the Jordanian and French Legislation
Source: F1000Res. 2026 Mar 5;15:359. [Version 1] doi: 10.12688/f1000research.177079.1 (PMC12993289; doi:10.12688/f1000research.177079.1)
Supplement: Peer review history

# Reviewer report: Reviewer response for version 1

**DOI:** 10.5256/f1000research.195235.r465805

**Published:** 2026-03-16

**Authors:** Hamza Abu Issa

**Affiliations:** 1 Applied Science Private University, Amman, Jordan

This article addresses a relevant and timely issue in Jordanian cybercrime law by examining the legal regulation of “character assassination” under Article 16 of the Jordanian Cybercrime Law No. 17 of 2023, with comparative reference to French legislation. The manuscript aims to clarify the concept of the offence, distinguish it from related offences such as defamation, libel, slander, and false information, and propose legislative reforms. The topic has clear academic merit, and the doctrinal legal approach adopted by the authors is appropriate for the subject. The article also identifies a genuine legislative ambiguity in the Jordanian provision and reaches conclusions that are generally consistent with the legal analysis presented.

 At the same time, the manuscript is only partly clear and accurate in its present form. It would benefit from language editing, clearer and more consistent terminology, and a more precise presentation of some comparative references and legal authorities. In addition, although the article states that it uses descriptive and legal analytical methods, the methodology section remains somewhat general. The paper would be strengthened if the authors explained more clearly the legal materials examined, the basis for selecting French law as the comparator, and the criteria used in conducting the comparison. These issues do not undermine the overall value of the study, but they should be addressed to improve clarity, transparency, and precision.

 No statistical analysis is involved, and no conventional dataset is required for this type of doctrinal legal research. However, the article would still benefit from a clearer identification of the principal legal sources underlying the analysis. In order to justify full approval, the authors should revise the language, standardize the terminology, strengthen the precision of the comparative discussion, expand the methodology section, and provide fuller citations for the main legal authorities relied upon. Subject to these revisions, the article can make a useful contribution to the study of cybercrime and reputational harm in Jordanian criminal law.

Is the work clearly and accurately presented and does it cite the current literature?

Partly

If applicable, is the statistical analysis and its interpretation appropriate?

Not applicable

Are all the source data underlying the results available to ensure full reproducibility?

No source data required

Is the study design appropriate and is the work technically sound?

Yes

Are the conclusions drawn adequately supported by the results?

Yes

Are sufficient details of methods and analysis provided to allow replication by others?

Partly

Reviewer Expertise:

Criminal Law

I confirm that I have read this submission and believe that I have an appropriate level of expertise to confirm that it is of an acceptable scientific standard.

## References

[1] : Character Assassination via Information Technology Media: A New Crime. 10.1007/978-3-031-90558-2_26 203-208 10.1007/978-3-031-90558-2_26

# Reviewer report: Reviewer response for version 1

**DOI:** 10.5256/f1000research.195235.r465809

**Published:** 2026-03-16

**Authors:** Abdullah M Ehjelah

**Affiliations:** 1 Yarmouk University, Irbid, Irbid Governorate, Jordan

**Abstract of the Article**

 The manuscript presents an interesting and relevant study in its field. The authors address an important research question and provide a clear overview of the topic supported by relevant literature. The objectives of the study are well defined, and the structure of the manuscript allows the reader to follow the research process and findings effectively.

**Comments on the Evaluation Criteria**

**Clarity and presentation of the work:**

 The article is clearly written and well organized. The introduction provides sufficient background and appropriately cites relevant and recent literature. Overall, the presentation of the research is coherent and understandable.

**Study design and technical soundness:**

 The study design appears appropriate for addressing the research objectives. The methodology used is suitable and reflects good scientific practice.

**Methods and analysis details:**

 The authors provide sufficient methodological details to allow other researchers to understand and potentially replicate the study. The description of the procedures and analytical steps is generally clear.

**Availability of source data:**

 The results presented in the manuscript are adequately supported by the data described. The level of information provided allows readers to understand how the conclusions were reached.

**Conclusions:**

 The conclusions are supported by the results and are consistent with the aims of the study. The discussion appropriately relates the findings to the broader literature.

**Overall Recommendation**

 Overall, this manuscript represents a valuable contribution to the field. The study is well conducted, clearly presented, and provides meaningful insights. I recommend acceptance of the article after minor editorial review if required.

 - **The researcher provides** a distinguished scientific treatment of a timely and important topic in Jordanian legislation, namely the crime of **character assassination** as stipulated in Article 16 of the Cybercrime Law No. 17 of 2023. The researcher successfully highlights the legal problem associated with the absence of a precise definition of this term in the statutory text, offering a clear analysis of the surrounding legal context and its relation to closely related offenses such as defamation, libel, and the dissemination of false information. This approach demonstrates the researcher’s ability to critically read and analyze legal texts, helping to clarify legislative ambiguities and identify potential practical challenges arising from this crime.

 The study is also distinguished by its use of a comparative analytical method. The researcher effectively employs comparisons with other legal systems, particularly the French legal framework, to illustrate how reputation-related offenses are addressed in other jurisdictions. This approach adds significant scholarly value to the research and deepens the legal understanding of character assassination in the contemporary digital environment, especially in light of the challenges posed by the rapid spread of information through the internet and social media platforms.

 The value of this research is further evident in the constructive critical analysis it provides of Jordanian legislation. The researcher does not merely present the statutory texts but evaluates them and identifies possible shortcomings, offering well-grounded recommendations to clarify legal concepts and distinguish between overlapping offenses that harm reputation. These observations and recommendations can contribute to the development of the legislative framework, enhance clarity in statutory texts, improve judicial application, and protect individual rights and societal interests. This reflects the researcher’s diligence and significant scholarly contribution to the field.

 However, to further enhance the study’s value and completeness, it would be beneficial for the researcher to address the following questions:

1. Is a personal claim or complaint from the victim required to initiate criminal proceedings against the perpetrator of the crime of character assassination?
2. Does the criminal law equate the penalty for character assassination with the penalties prescribed for defamation and libel committed via information networks?
3. Is it legally conceivable for the crime of character assassination to be committed against a legal entity, or is its scope limited to natural persons only?
4. What is the legal meaning of the terms used in Article 16, such as “spread,” “attributed,” and “assigned deliberately,” and how do they differ in legal significance?
5. In the researcher’s opinion, is there a need to enact a separate legal provision specifically criminalizing character assassination, given the existence of clear and comprehensive provisions addressing defamation, libel, and insults committed via information networks?
6. Is it legally conceivable to allow installment payments for the financial penalty imposed for character assassination if confirmed by a final court judgment?
7. Is it possible to replace the custodial penalty prescribed for character assassination with a community-based alternative under Jordanian criminal law?
8. It is recommended to enrich the reference list with recent and up-to-date sources relevant to the field of the study to strengthen the research and support the findings.

Is the work clearly and accurately presented and does it cite the current literature?

Yes

If applicable, is the statistical analysis and its interpretation appropriate?

I cannot comment. A qualified statistician is required.

Are all the source data underlying the results available to ensure full reproducibility?

Yes

Is the study design appropriate and is the work technically sound?

Yes

Are the conclusions drawn adequately supported by the results?

Yes

Are sufficient details of methods and analysis provided to allow replication by others?

Yes

Reviewer Expertise:

Criminal Law, Cybercrime Law

I confirm that I have read this submission and believe that I have an appropriate level of expertise to confirm that it is of an acceptable scientific standard.
